# International Classification of Functioning in professional rehabilitation: instruments for assessing work disability

**DOI:** 10.11606/s1518-8787.2020054001463

**Published:** 2020-04-28

**Authors:** Juliana Scholtão Luna, Gina Torres Rego Monteiro, Rosalina Jorge Koifman, Anke Bergmann

**Affiliations:** I Universidade Federal do Acre Diretoria de Saúde e Qualidade de Vida do Servidor Federal Rio BrancoAC Brasil Universidade Federal do Acre . Diretoria de Saúde e Qualidade de Vida do Servidor Federal . Rio Branco , AC , Brasil; II Fundação Oswaldo Cruz Escola Nacional de Saúde Pública Departamento de Epidemiologia e Métodos Quantitativos em Saúde Rio de JaneiroRJ Brasil Fundação Oswaldo Cruz . Escola Nacional de Saúde Pública . Departamento de Epidemiologia e Métodos Quantitativos em Saúde . Rio de Janeiro , RJ , Brasil; III Instituto Nacional de Câncer Programa de Epidemiologia Clínica Rio de JaneiroRJ Brasil Instituto Nacional de Câncer (INCA). Programa de Epidemiologia Clínica . Rio de Janeiro , RJ , Brasil

**Keywords:** Disability Assessment, Occupational Health, International Classification of Functioning, Disability and Health, Systematic Review

## Abstract

**OBJECTIVE:**

To review the main instruments of functional assessment and health status cited in the literature to evaluate Brazilian workers and verify the compatibility of their items with the core set for professional rehabilitation.

**METHODS:**

A review of the literature was conducted in the main databases in search of articles that used assessment instruments in populations of workers between 2007 and 2017. Subsequently, the contents of the identified instruments were retrieved, and two evaluators analyzed their items to verify the compatibility with the categories of the core set of the International Classification of Functioning for professional rehabilitation. Cohen’s kappa coefficient was used to evaluate the agreement between the evaluators.

**RESULTS:**

Five specific and eight generic instruments were selected to evaluate the functioning of workers. The analysis of the items of the total instruments allowed the definition of 58 categories (64.5%) of the core set with minimal overlap: 13 (76.5%) of the body functions component, 29 (72.5%) of the activities and participation component and 16 (49%) environmental factors.

**CONCLUSIONS:**

The association of several instruments requires time and makes it difficult to use the classification. The development of instruments with direct association with its categories is essential to operationalize it.

## INTRODUCTION

The use of classification instruments in the area of occupational health facilitates the understanding of different work-related injuries and disabilities ^1 , 2^ , since its consequences can vary. The standardization of language on the health status of workers optimizes the relationships between the various areas that make up the intervention team, enabling integrated and effective actions to return to work ^1^ .

The World Health Organization (WHO) proposes two classifications to record information on the health conditions and states of populations. The first is the International Statistical Classification of Diseases and Related Health Problems (ICD), which codifies health conditions in terms of signs and symptoms. The second is the International Classification of Functioning, Disability and Health (ICF), which classifies the impact of these conditions in terms of functioning ^4^ . These classifications should be used jointly, favoring comparisons in research, data records, as well as feeding information systems and reporting statistics in public health ^5 , 6^ .

Professional rehabilitation (PR) is described as the main process to promote workers’ health, aiming to keep the worker active or providing their return to work in cases of illness or disability ^1 , 7 , 8^ . It should be carried out under a interprofessional approach, with actions ranging from disease prevention and health care to changes in the work environment ^1 , 2 , 9^ .

In Brazil, PR is historically attributed to the Ministry of Social Security, having as its central criterion the identification of disability through expertise with subsequent granting of benefit to the worker on leave and referral to a professional guidance program that aims to promote means (training and courses) for the worker to be reinserted in the labor market ^10^ . It does not necessarily include integrated physical and/or psychological rehabilitation actions, which are the tasks of the Ministry of Health, offered by rehabilitation centers, not necessarily specialized in this area of activity ^10 , 11^ .

The Technical Manual of Procedures in the Area of Professional Rehabilitation, 2016, points to an advance in this area, recognizing that PR should include combined actions of care, surveillance and health care, including social reintegration and environmental analysis, with the performance of a specialized multidisciplinary team of public and intersectoral responsibility and guided by ICF throughout its preparation ^12^ . However, the disarticulation between the responsible sectors and the lack of a consistent public policy still contribute to the lack of implementation of an efficient PR program in Brazil, with many workers unable to return to their work activities ^10^ .

The ICF Research Branch is an important WHO collaborating center for studies with ICF and is a reference in PR publications. The adoption of the WHO biopsychosocial model, as well as the use of ICF in PR, are already consolidated by allowing a comprehensive view of work disability and language standardization, improving communication between health professionals, users, employers and public policy managers, favoring the expected results with PR ^13^ .

In this sense, a group of researchers from this center elaborated in 2012 the core set for PR, to serve as a reference in the description of worker functioning ^2^ . The core set is a short list of categories of ICF, elaborated by expert consensus in a given area, as a strategy proposed by the WHO to facilitate the use of classification among professionals from the various sectors ^14^ .

The core set for PR brings together 90 categories of ICF and is applicable to any class of workers, regardless of health conditions. It is divided in 17 categories related to body functioning, 40 categories regarding activities and participation and 33 related to environmental factors ( Table 1 ).

**Table 1:** Categories of the core set of the International Classification of Functioning, Disability and Health for professional rehabilitation according to Finger et al. 12

Body functions (b)		Activity and participation		Environmental factors (e)	
		
ICF category	Category description	ICF category	Category description	ICF category	Category description
b 117	Intellectual functions	d 155	Skill acquisition	e 1101	Medication
b 126	Temperament and personality	d 160	Focus	e 115	Products for personal use in daily life
b 130	Energy and impulse functions	d 163	Thinking	e 120	Personal mobility products
b 134	Sleep functions	d 166	Reading	e 125	Products and technologies for communication
b 140	Attention-related functions	d 170	Writing	e 130	Products and technologies for education
b 144	Memory functions	d 172	Mathematical thought	e 135	Products and technology for employment
b 152	Emotional functions	d 175	Problem-solving	e 150	Architecture, public use construction
b 160	Thought functions	d 177	Decision-making	e 155	Architecture, private use construction
b 164	High-level cognitive functions	d 210	Accomplishing a single task	e 225	Climate
b 210	Vision-related functions	d 220	Perform multiple tasks	e 240	Light
b 230	Hearing-related functions	d 230	Accomplishment of daily routine	e 250	Sound
b 235	Vestibular functions	d 240	Dealing with stress and other psychological demands	e 260	Air quality
b 280	Feeling of pain	d 310	Communication and reception of verbal messages	e 310	Immediate family
b 455	Tolerance to physical exercise	d 315	Communication and reception of nonverbal messages	e 320	Friends
b 730	Muscle strength functions	d 350	Talking	e 325	Acquaintances, peers, colleagues, neighbors
b 740	Muscular endurance functions	d 360	Use of communication devices	e 330	Authority figures
b 810	Skin protection functions	d 410	Changing the basic position of the body	e 340	Personal caretakers
		d 415	Maintain body position	e 355	Healthcare professionals
		d 430	Lifting and transporting objects	e 360	Other professionals
		d 440	Use fine hand movements	e 430	Attitude of authority figures
		d 445	Use of hand and arm movements	e 450	Individual attitudes of health professionals
		d 450	Walking	e 460	Social attitudes
		d 455	Moving	e 465	Practical norms and ideologies
		d 465	Move using equipment	e 525	Housing services, systems and policies
		d 470	Transportation	e 535	Communication services and policies
		d 475	Conducting of vehicles	e 540	Transport services and policies
		d 530	Care towards the excretion process	e 550	Legal services, systems and policies
		d 540	Dressing	e 555	Associations services and policies
		d 570	Taking care of one’s own health	e 565	Economic services, systems and policies
		d 710	Basic interpersonal interactions	e 570	Social security services and policies
		d 720	Complex personal interactions.	e 580	Healthcare services, systems and policies
		d 740	Formal relationship	e 585	Education-related services and policies
		d 820	School education	e 590	Labor and employment services and policies
		d 825	Professional Training		
		d 830	Higher education		
		d 840	Internship/preparation for work		
		d 845	Acquiring, keeping and leaving a job		
		d 850	Paid job		
		d 855	Unpaid job		
		d 870	Economic self-reliance		

It is important to note that ICF and its core sets are merely classification instruments and in order for them to be accessed in a legitimate way with its findings being subject to comparison by peers, standardized methods are required to evaluate the functioning of different individuals, preferably compatible with the use of the classification ^14^ . In order to contribute to the operationalization of ICF, essential for the advancement of public PR policies in Brazil, this study aimed to review the instruments of functional assessment and health status that are used in the literature regarding functioning and health status of Brazilian workers in general and to verify the compatibility of their items with the categories of the core set for professional rehabilitation.

## METHODS

A literature review was conducted in the PubMed, Scopus, Web of Science, Lilacs and SciELO databases *,* from January to July 2018 *,* with the objective of identifying instruments for assessing the functioning and health status applied to populations of Brazilian workers of any category. The literature search included publications from January 2007 to December 2017.

The following descriptors were considered: “capacity evaluation, work,” OR “disability evaluation, work,” AND “functional assessment,” OR “questionnaire.” The same descriptors were used for searching in Portuguese.

After the identification of the articles, the instruments used in the studies for the evaluation of workers were selected, and their contents were searched in full. Then, we selected those in which most items could respond to the categories of the core set of ICF for PR, including, preferably, those with items related to the evaluation of changes in body functions, limitation of activities and environmental issues, as proposed by the classification. From this, the analysis of the items of each instrument selected was made and the possibility of accessing the categories of the core set of ICF for PR was verified.

The compatibility of each item with the core set of the instruments identified in the literature *,* was independently verified by two evaluators, health professionals familiar with the use of ICF, as recommended in the literature ^15^ . For this analysis, it was considered what each item contemplated and thus established the connection with the core set through the detailed description and definitions of each category offered by ICF.

To verify the agreement between the evaluators in the selection of the categories used by the items of each instrument, Cohen’s kappa coefficients were calculated, classified by the cutoff points proposed by Landis and Koch ^16^: below 0 (poor); 0 to 0.20 (weak); 0.21 to 0.40 (reasonable); 0.41 to 0.60 (moderate); 0.61 to 0.80 (substantial); and 0.81 to 1.00 (almost perfect).

After the analysis of the agreement, the non-concordant items were studied among the evaluators in search of consensus for either the removal or the inclusion of the item as compatible with the core set.

## RESULTS

We found 13 assessment instruments used in research on functioning or health status of Brazilian workers in the period studied. Through the recovery of their contents, it was found that all were validated specifically for Brazil to evaluate aspects related to functioning or health status.

Between the thirteen reviewed instruments, five were created specifically for the evaluation of disabilities and work-related aspects, including: the Work Ability Index (WAI), the Cultural and Psychosocial Influences on Disability questionnaire (CUPID), the Work Disability Diagnosis Interview (WoDDI), the Obstacles to Return-to-Work Questionnaire (ORTWQ) and the Work Role Functioning Questionnaire (WRFQ). The others were instruments used by the researchers, on groups of workers, but created for specific evaluation of some anatomical regions or to verify quality of life in general without taking into account the nature and working conditions that may be associated with symptoms. The instruments analyzed and the number of categories of the core set for PR that could be accessed by each of them are presented in Table 2 .

**Table 2:** Functional assessment and health status instruments selected for compatibility analysis with the core set of the International Classification of Functioning, Disability and Health for professional rehabilitation and number of categories of the core set that could be accessed in each domain.

Instrument	Author, year and place of study	Sample of workers	Number of categories accessed		

			Body functions Total: 17	Activity and participation Total: 40	Environmental factors Total: 33
WAI	Walsh et al., 2008. São Carlos, SP.	134 workers of a multinational company	3	9	0
CUPID	Carugno et al., 2012. São Paulo, SP.	751 nurses from public hospitals	3	7	1
WoDDI	Mininel et al., 2012. São Paulo, SP.	30 workers of the University Hospital of USP	6	6	5
WRFQ	Galash and Costa, 2007. Campinas, SP.	105 workers (formal or informal)	1	14	0
ORTWQ	Milani et al., 2016. Campinas, SP.	301 miscellaneous workers	3	2	4
WHODAS	Valério et al., 2016. Uberaba, MG.	94 active workers (formal or informal)	2	13	1
NHP	Bartilotti et al., 2009. Florianópolis, SC.	425 workers assisted at CEREST in Santa Catarina	5	7	1
FIM	Bartilotti et al., 2009. Florianópolis, SC.	425 workers assisted at CEREST in Santa Catarina	1	8	0
DASH.	Camargo et al., 2007. São Carlos, SP.	27 industrial workers	4	5	0
ODI	Walsh et al., 2008. São Carlos, SP.	134 workers of a multinational company	2	6	0
RMQ	Sardá Jr et al., 2009. Florianópolis, SC.	234 refrigerator workers	3	7	01
SF-36	Sena et al., 2013. Lagarto, SE.	351 rural workers	3	10	0
WHOQOL-BREF	Ferreira et al., 2017. São Paulo, SP.	50 butchers	4	6	11

WAI; Work Ability Index; CUPID: Cultural and Psychosocial Influences on Disability questionnaire; WoDDI: Work Disability Diagnosis Interview; WRFQ: Work Role Functioning Questionnaire; ORTWQ: Obstacles to Return-to-Work Questionnaire; WHODAS: World Health Organization Disability Assessment Schedule II; NHP: Nottingham Health Profile; FIM: Functional Independence Measure; DASH: Disabilities of the Arm, Shoulder and Hand questionnaire; ODI: Oswestry Low Back Pain Disability Questionnaire; RMQ: Roland-Morris Questionnaire; SF-36: Short Form (36) Health Survey; WHOQOL-BREF: short version of the World Health Organization Quality of Life questionnaire; USP: Universidade de São Paulo; CEREST: *Centro de Referência em Saúde do Trabalhador* (Reference Center in Occupational Health)

Among these instruments, four were developed based on ICF categories, which allows for a greater connection with said index: the World Health Organization Disability Assessment Schedule II (WHODAS), the Nottingham Health Profile (NHP), the World Health Organization Quality of Life questionnaire (WHOQOL) and the ORTWQ. The WHODAS showed substantial agreement between the evaluators in the link with the core set, and the others almost perfect agreement, as shown in Table 3 . The other eight instruments reviewed were not created in order to be used with ICF, but the agreement between the items chosen by the evaluators as compatible with the categories of the core set was substantial or almost perfect for most and moderate for the CUPID ( Table 3 ).

**Table 3:** Instruments analyzed and kappa values found in the analysis of agreement between the evaluators.

Instrument	Kappa value
WRFQ	0.91
NHP	0.87
ODI	0.86
ORTWQ	0.83
DASH.	0.82
WHOQOL-BREF	0.81
SF-36	0.78
WHODAS	0.74
RMQ	0.73
FIM	0.72
WAI	0.67
WoDDI	0.61
CUPID	0.49

WRFQ: Work Role Functioning Questionnaire; NHP: Nottingham Health Profile; ODI: Oswestry Low Back Pain Disability Questionnaire; ORTWQ: Obstacles to Return-to-Work Questionnaire; DASH: Disabilities of the Arm, Shoulder and Hand questionnaire; WHOQOL-BREF: short version of the World Health Organization Quality of Life questionnaire; SF-36: Short Form (36) Health Survey; WHODAS: World Health Organization Disability Assessment Schedule II; RMQ: Roland-Morris Questionnaire; FIM: Functional Independence Measure; WAI: Work Ability Index; WoDDI: Work Disability Diagnosis Interview; CUPID: Cultural and Psychosocial Influences on Disability questionnaire

Note: Classification according to Landis and Koch ^16^: poor (<0), weak (0 to 0.20), reasonable (0.21 to 0.40), moderate (0.41 to 0.60), substantial (0.61 to 0.80), almost perfect (0.81 to 1).

**Graph 1:**
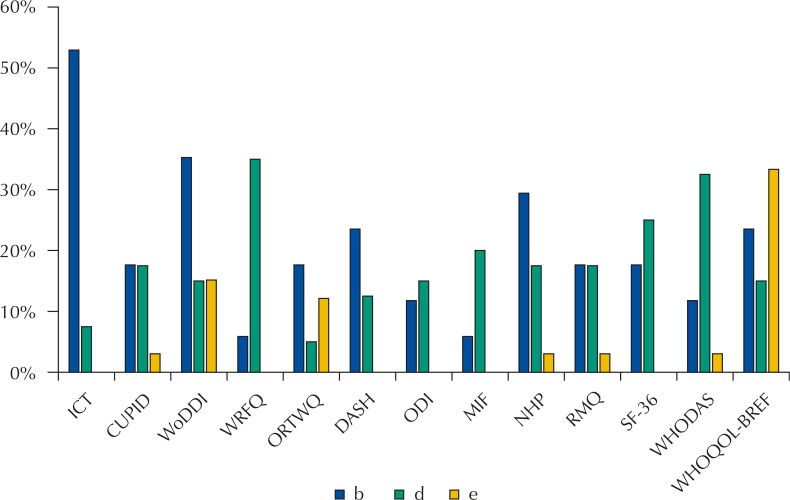
Proportion of categories filled by the instruments analyzed in each domain of the core set for professional rehabilitation. WAI; Work Ability Index; CUPID: Cultural and Psychosocial Influences on Disability questionnaire; WoDDI: Work Disability Diagnosis Interview; WRFQ: Work Role Functioning Questionnaire; ORTWQ: Obstacles to Return-to-Work Questionnaire; DASH: Disabilities of the Arm, Shoulder and Hand questionnaire; ODI: Oswestry Low Back Pain Disability Questionnaire; FIM: Functional Independence Measure; NHP: Nottingham Health Profile; RMQ: Roland-Morris Questionnaire; SF-36: Short Form (36) Health Survey; WHODAS: World Health Organization Disability Assessment Schedule II; WHOQOL-BREF: short version of the World Health Organization Quality of Life questionnaire.

The analysis of the compatibility of the selected instrument items with the core set for PR highlighted the variability in the focus of each instrument when addressing functioning issues of the individual when the objective is occupational capacity. The proportion of categories in the *core set* accessed by the items of each questionnaire according to the components of ICF is presented in Graph 1 and the emphasis given to the evaluation of body functions and limitation of activities and participation is noted. Specific instruments for evaluating work-related aspects (WAI, CUPID, WoDDI, WRFQ and ORTWQ), including functioning limitations, were able to access, when analyzed together, 36 categories of the core set for professional rehabilitation (11 categories b, 15 categories d and 10 categories e).

WAI aims to highlight how well a worker is or will be in the near future and how well he is able to perform his work according to his demands, his health status and his physical and mental abilities. It includes aspects such as current capacity for work, capacity in relation to work requirements, number of diagnosed diseases, estimated loss of work ability and absence due to diseases, which is prognosis of work ability two years in the future as well as mental resources. From its items it was possible to connect it with 12 categories of the core set in question.

CUPID aims to associate the musculoskeletal symptoms of workers with their activities, psychosocial aspects and other disabilities. Validated for Brazil as an International Survey on Physical, Cultural and Psychosocial Influences on Musculoskeletal Symptoms and Associated Disabilities, it verifies physical activities at work, psychosocial aspects, musculoskeletal symptoms in various anatomical sites associated with disability for common daily tasks, mental health and tendency to worry about symptoms, as well as beliefs about the nature and severity of work-related diseases. This instrument allowed for a connection with 11 categories.

WoDDI seeks to detect the most important predictive factors for work-related disabilities and to identify one or more causes of prolonged absenteeism. Validated for Brazil as an Instrument for Identifying the Situation of Disability at Work, it analyzes the history of current illness, pain, previous and current health condition, physical examination, life habits, socio-family history, financial situation, work environment, worker perceptions and analysis of results and recommendations. These items were compatible with 17 categories of the core set.

WRFQ is validated for the Portuguese language as a Work Performance Assessment Questionnaire and assesses whether the worker’s functional capacity is altered due to health problems. It analyzes the work-related, physical, mental, social and production-related demands. It was connected to 15 categories.

The last instrument used for evaluation of workers, ORTWQ, was created under the influence of ICF and evaluates barriers related to return to work. Validated for Brazil as Obstacles for the Return to Work, it contains 55 items divided into nine domains: difficulty of return, physical load and self-perception of harmfulness in work, social support, concern due to absence, satisfaction, family support or situation, and self-perceived prognosis of return to work. Although extensive, the analysis of these items allowed for a connection with only nine categories of the core set.

The other reviewed instruments were adapted to propose different problems for the workers and, when linked to the core set for professional rehabilitation, also presented variability within the categories answered, with emphasis on body functions and limitation of activities. When used in conjunction, these instruments were able to access a total of 45 categories, with seven being related to body functions, 24 to activities and participation and 13 to environmental factors.

WHODAS was created by the World Health Organization to provide direct correlation with ICF and serve as a standardized method for measuring health and disabilities in a cross-cultural way. It contains 36 items and provides level of functioning in six domains: cognition, mobility, self-care, interpersonal relationships, life activities and participation. From its items it was possible to connect it with 16 categories of the core set in question.

NHP is a generic instrument with 38 items that refer to the following aspects: energy level, pain, emotional reactions, sleep, social interaction and physical abilities. It aims to evaluate the quality of life in patients with chronic diseases. The analysis of its items showed compatibility with 13 categories.

The Functional Independence Measure (FIM), validated for the Portuguese language, was elaborated based on the categories of the test version of ICF called International Classification of Impairments, Disabilities, and Handicaps (ICIDH). Validated for a wide category of people in Brazil, it measures the level of disability of the individual and how much assistance is required to perform his or her activities. It has 18 items distributed in the following domains: self-care, sphincter control, mobility, locomotion, communication and social interaction. It was compatible with 18 categories of the core set *.*

Another instrument used in the evaluation of functioning in workers is the Disabilities of the Arm, Shoulder and Hand questionnaire (DASH), created to assess upper limb dysfunction and physical symptoms in any group of people. It includes 30 items related to pain, weakness, stiffness, tingling, daily activities, household chores, shopping, recreational activities, self-care, dressing, eating, sexual activities, sleeping, family care, work, socialization, and self-image. Regarding the core set, the DASH allowed for the access of nine categories.

The Oswestry Low Back Pain Disability Questionnaire (ODI) and the Roland-Morris Questionnaire (RMQ) are also used for functional capacity assessment. Both validated for Brazil in several population types, they evaluate the effect of lower back pain on functionality. They were connected to 8 and 11 categories of the core set, respectively.

The last two reviewed instruments evaluated the quality of life of individuals, considering functional aspects. The Short Form (36) Health Survey (SF-36) assesses the quality of life of individuals and includes questions related to functioning. It is a generic form, and addresses the following aspects: functional capacity, physical aspects, pain, general health status, vitality, social aspects, emotional aspects, mental health and comparative health assessment. It allowed for connections with 13 categories.

WHOQOL-BREF also assesses quality of life. It is a reduced version of WHOQOL, proposed by the WHO to be used in a cross-cultural way, composed of 26 items distributed in four domains: physical, psychological, social relations and the environment. It allowed for the connection to 21 categories.

According to this present study, it would be necessary to use a combination of instruments to evaluate all aspects considered relevant pointed out in the categories of core set for professional rehabilitation. Completing the maximum categories of the core set using the least possible instruments would require the use of 10 of the 13 reviewed in this study and it would be possible to access 58 of the 90 proposed categories ( Table 4 ). Among them, 13 (76%) would refer to the component of body functions, 28 (72.5%) activities and participation and 16 (49%) environmental factors.

**Table 4:** List of the categories of the core set for professional rehabilitation accessed by the ten assessment instruments selected as the minimum to respond to the core set.

Categories*	Instruments
b 126: temperament functions	RMQ, DASH, WRFQ
b 130: energy and impulse functions	WAI, ORTWQ, SF-36, WHOQOL-BREF
b 134: sleep functions	WAI, WoDDI, RMQ, DASH, WHOQOL-BREF
b 140: attention-related functions	WAI
b 144: memory functions	WHODAS
b 152: emotional functions	CUPID, WAI, ORTWQ, WoDDI, SF-36, WHODAS, WHOQOL-BREF
b 210: vision-related functions	WAI
b 230: hearing-related functions	WAI
b 235: vestibular functions	CUPID
b 280: feeling of pain	CUPID, WAI, ORTWQ, WoDDI, RMQ, DASH, SF-36, WHOQOL-BREF
b 455: tolerance to physical exercise	WAI, WoDDI
b 730: muscle strength functions	WoDDI, DASH
b 810: skin protection functions	WAI, WoDDI
d 160: focus	WHODAS, WHOQOL-BREF, WRFQ
d 163: thinking	WRFQ
d 166: reading	WRFQ
d 170: writing	CUPID, DASH
d 175: problem-solving	WAI, WHODAS
d 177: decision-making	WAI
d 210: accomplishing a single task	ORTWQ, WHODAS, WRFQ
d 220: performing multiple tasks	RMQ
d 230: accomplishment of daily routine	CUPID, RMQ, SF-36, WHODAS, WHOQOL-BREF
d 310: communicating (verbal messages)	WHODAS, WRFQ
d 350: conversation	WHODAS
d 360: use of communication devices	WRFQ
d 410: changing the basic position of the body	CUPID, RMQ, SF-36, WHODAS, WRFQ
d 415: maintain body position	RMQ, WHODAS, WRFQ
d 430: lifting and transporting objects	WoDDI, DASH, SF-36, WRFQ
d 440: use fine hand movements	DASH, CUPID, WRFQ
d 445: use of the hand and arm	DASH, CUPID, ORTWQ, SF-36, WRFQ
d 450: walking	CUPID, WoDDI, RMQ, SF-36, WHODAS, WHOQOL-BREF, WRFQ
d 455: moving	SF-36
d 465: moving with equipment	WoDDI, RMQ
d 470: use of transportation	DASH
d 530: care towards the excretion process	FIM
d 540: dressing	CUPID, RMQ, DASH, SF-36, WHODAS
d 570: taking care of one’s own health	WHODAS
d 710: basic interpersonal interactions	SF-36, WHODAS, WHOQOL-BREF
d 740: formal relationship	WoDDI, WRFQ
d 845: acquiring, keeping and leaving a job	SF-36
d 850: paid work	WAI, WoDDI, SF-36, WHODAS, WHOQOL-BREF, WRFQ
d 870: economic self-reliance	WoDDI, WHOQOL-BREF
e 1101: medication	WoDDI
e 120: mobility products	RMQ
e 150: architecture (public buildings)	WHODAS
e 225: climate	WoDDI, WHOQOL-BREF
e 240: light	WHOQOL-BREF
e 250: sound	WoDDI, WHOQOL-BREF
e 260: air quality	WoDDI, WHOQOL-BREF
e 310: immediate family	ORTWQ, WHOQOL-BREF
e 320: friends	ORTWQ, WHOQOL-BREF
e 325: acquaintances	ORTWQ, WoDDI, WHOQOL-BREF
e 330: authority figures	CUPID
e 355: healthcare professionals	WHOQOL-BREF
e 430: attitude of authority figures	ORTWQ
e 525: housing-related services	WHOQOL-BREF
e 525: transportation-related services	WHOQOL-BREF
e 580: health services	WoDDI, WHOQOL-BREF

WRFQ: Work Role Functioning Questionnaire; NHP: Nottingham Health Profile; ODI: Oswestry Low Back Pain Disability Questionnaire; ORTWQ: Obstacles to Return-to-Work Questionnaire; DASH: Disabilities of the Arm, Shoulder and Hand questionnaire; WHOQOL-BREF: short version of the World Health Organization Quality of Life questionnaire; SF-36: Short Form (36) Health Survey; WHODAS: World Health Organization Disability Assessment Schedule II; RMQ: Roland-Morris Questionnaire; FIM: Functional Independence Measure; WAI: Work Ability Index; WoDDI: Work Disability Diagnosis Interview; CUPID: Cultural and Psychosocial Influences on Disability questionnaire

Note: 32 categories of the core set, described in Table 1, were not identified in any instrument, four of which were body-related functions (b117, b160, b164, b740), 11 activities and participation (d155, d172, d240, d315, d475, d720, d820, d825, d830, d840, d855) and 17 environmental factors (e115, e125, e130, e135, e155, e340, e360, e450, e460, e465, e535, e550, e565, e555, e570, e585, e590).

Most instruments prioritize the evaluation of changes in body functions and the identification of limitation of activities that may interfere with work, as they were analyzed to work with the core set for professional rehabilitation. Environmental factors are considered by six instruments; therefore, environmental interference as a facilitator or barrier is poorly addressed by most of the instruments raised.

## DISCUSSION

This study aimed to analyze instruments for the assessment of functioning to access the core set of ICF for PR, aiming to improve the understanding functioning measures in this area and elucidate possible ways of using the classification. This core set was chosen because it is the only one in the literature created to guide the classification of the functioning of workers in rehabilitation, through ICF. To use it, it is important to apply validated instruments that reliably measure functioning, generating results that can be compared in research ^2^ .

The review conducted during the research showed the multiplicity of instruments used in the approach of functional issues related to work. Those who could reach ICF domains were eligible, accounting for the maximum number of categories in the core set studied. Thus, five specific instruments and eight generic instruments were selected that evaluated functioning published in the proposed period.

The wide variety of instruments available, with different theoretical bases, leads to the need for decisions on the part of researchers and makes it difficult to compare and standardize the results ^17^ . One way to compensate for this gap is to link different assessment instruments to a single conceptual model that favors a common framework for comparing measures ^18^ .

ICF is a WHO-endorsed theoretical conceptual basis designed to classify functioning and disability of individuals and promote a unique and comprehensive language about the health status of different populations ^21 , 22^ . It is linked to evaluation instruments, and as such it allows the translation of these measures into the same language (that is, ICF codes), facilitating the content analysis of its items and the understanding and comparison of the results ^23^ .

Analyzed based on the core set for PR, it was possible to notice that the instruments gather common items but differ in the approach of the domains considered by ICF. About 38% of the categories in the core set could be accessed by more than one of the 13 instruments. In relation to the domains, the components related to problems in body functions, limitation of activities and participation were the most represented, evidencing the concern to verify physical illness and the experience of workers in the various activities involved.

A total of 32 of the 90 categories of the core set could not be accessed through the items of the instruments found. The body functions component had four unanswered categories (23%), the one related to activities and participation had four unanswered categories (27.5%) and the least represented component was the one related to environmental factors, with 17 unanswered categories (51.5%).

Other authors also verified this lower representativeness of items related to the categories of environmental factors of ICF in correlations with other instruments ^17 , 19 , 24^ . As such, this fact disfavors the approach given to functioning, since it ignores an important influencing component in the functioning of individuals, highly considered by the biopsychosocial model that supported ICF ^23 , 27^ .

In PR, environmental factors (be they physical, social and attitudinal) should be emphasized, since they are important influencing factors in work participation, interacting with body conditions (functions and body structures) and determining the level and extent of its functioning ^1 , 28^ . The main objective of this process is the recovery of labor capacity in an effective and lasting manner, which is closely associated with workplace conditions ^3^ .

To remain active and productive, the worker needs to be in a favorable environment; which must include facilitators of functioning, from adequate ergonomic conditions to organizational changes that generate well-being, increased self-esteem, autonomy at work and healthy relationships ^1 , 29^ . Even highly disabled individuals can have their possible participation recovered if the modification of the environment is among the priorities of PR ^1 , 3^ . This process occurs with the analysis of environmental factors that must be carefully inserted in the evaluation and monitoring of each worker ^3^ .

The kappa coefficient, used to verify the agreement between the evaluators who analyzed the correspondence of the items of the instruments with the categories of the core set, ranged from moderate agreement (k = 0.49) for an instrument to almost perfect (k > 0.80) for seven instruments ( Table 3 ). This result indicates that, although the instruments do not offer a direct link with ICF, the evaluators had a common understanding in choosing most of the categories accessed by each item.

Similar results have been found in other studies in the connection of ICF with different tools, and the authors point out that disagreement between the evaluators may be due to ICF presenting more specific categories in some areas than in other ^19 , 22 , 27^ . In addition, the interpretation of a given concept in an item of an instrument may differ among evaluators, in such a way that distinct categories of the core set could be selected; this justifies the need for consensus in the final choice of categories accessed by the questionnaires ^30^ . The link of assessment instruments with ICF allows a standardized analysis of its contents, favoring the choice of the most appropriate instrument for use in clinical practice ^25 , 30^ .

According to this study, the evaluation of all aspects considered relevant in the worker’s functionality, suggested in the core set for professional rehabilitation, would require the combination of several instruments and the search for more forms of evaluation to include the missing items, since not all categories were contemplated. This is an acceptable result, since the core sets are instruments created by a consensus-based methodology among experts who seek to gather categories of ICF aimed at specific groups of the population, based on knowledge and clinical experience in the area, without taking into account the assessment instruments available to access them ^31^ .

For ICF and its core sets to be used more conveniently and uniformly, it is recommended to create measurement instruments attuned to the classification ^34^ . For this, the authors of this core set created the Work Rehabilitation Questionnaire (WORQ) ^34^ , adapted for Brazil as a Rehabilitation Questionnaire for Work, which can be used to evaluate several categories, providing a direct link with ICF.

This study is innovative when analyzing a considerable number of instruments that have been applied in the evaluation of the functioning of workers and verifying their approaches using as reference an ICF core set specific to this area of activity. The fact that not all instruments available for functional evaluation including physical or psychic aspects that can be used with workers were analyzed does not compromise the study result, since its objective was to illustrate the operationalization of ICF through this specific core set, prioritizing instruments that could respond to the largest possible number of categories.

In this sense, it was observed that it was not possible to establish linkage of items with all categories of the core set when instruments created without the purpose of using the classification are used. Further studies are needed to verify the degree of compatibility between how each tool quantifies the magnitude of the evaluated commitment and the technique adopted by ICF through its qualifiers, a fact that can constitute another barrier for the classification of the functioning evaluated from these instruments.

In addition, the link to the same conceptual basis allowed the visualization of the common aspects among the instruments studied, as well as the differences in the way of approaching the functioning or health status of individuals.

It was clear that environmental factors are still highly disregarded by functioning evaluation models used in research with workers. This is negative when it aims to restore work capacity and reinsert the individual in his or her workplace, because environmental factors are determinant for the effectiveness of actions and maintenance of functioning. It is necessary to include the evaluation of the environment in the PR process, and this can be done with the use of ICF, taking as reference the environmental categories suggested by the core set studied.

## CONCLUSION

The review of instruments for assessing the functioning and health status of workers and the subsequent link with the core set for professional rehabilitation performed by this study concluded that at least ten instruments would be necessary to evaluate 65% of the aspects considered relevant in the categories of the core set for professional rehabilitation. The component related to environmental factors in the core set was the one that presented the lowest possibility for answers through the items of the studied questionnaires, which indicates the minor emphasis given to these factors in disabilities. Associating multiple instruments to respond to a specific core set requires time and makes it difficult to use the classification. Evaluation instruments designed to allow direct association with ICF categories, and its qualifiers are essential to operationalize it.
