# Supplementary material for: International Classification of Functioning in professional rehabilitation: instruments for assessing work disability
Source: Rev Saude Publica. 2020 Apr 28;54:45. doi: 10.11606/s1518-8787.2020054001463 (PMC7190096; doi:10.11606/s1518-8787.2020054001463)
Supplement: Translation

# Translation: Classificação Internacional de Funcionalidade na reabilitação profissional: instrumentos de avaliação da incapacidade laboral

**Authors:** Juliana Scholtão Luna, Gina Torres Rego Monteiro, Rosalina Jorge Koifman, Anke Bergmann

**Affiliations:** I Brasil Universidade Federal do Acre. Diretoria de Saúde e Qualidade de Vida do Servidor Federal. Rio Branco, AC, Brasil; II Brasil Fundação Oswaldo Cruz. Escola Nacional de Saúde Pública. Departamento de Epidemiologia e Métodos Quantitativos em Saúde. Rio de Janeiro, RJ, Brasil; III Brasil Instituto Nacional de Câncer (INCA). Programa de Epidemiologia Clínica. Rio de Janeiro, RJ, Brasil

**Keywords:** Avaliação da Deficiência, Saúde do Trabalhador, Classificação Internacional de Funcionalidade, Incapacidade e Saúde, Revisão Sistemática

## Abstract

**OBJETIVO:**

Revisar os principais instrumentos de avaliação funcional e situação de saúde citados na literatura para avaliar trabalhadores brasileiros e verificar a compatibilidade de seus itens com o *core set* para reabilitação profissional.

**MÉTODOS:**

Foi realizada uma revisão da literatura nas principais bases de dados em busca de artigos que utilizaram instrumentos de avaliação em populações de trabalhadores entre 2007 e 2017. Posteriormente foram recuperados os conteúdos dos instrumentos identificados e dois avaliadores analisaram seus itens para verificar a compatibilidade com as categorias do *core set* da Classificação Internacional de Funcionalidade para reabilitação profissional. O coeficiente kappa de Cohen foi utilizado para avaliar a concordância entre os avaliadores.

**RESULTADOS:**

Foram selecionados cinco instrumentos específicos e oito genéricos que avaliaram a funcionalidade de trabalhadores. A análise dos itens do total de instrumentos permitiu o preenchimento de 58 categorias (64,5%) do *core set* com o mínimo de sobreposição: 13 (76,5%) do componente funções corporais, 29 (72,5%) do componente de atividades e participação e 16 (49%) de fatores ambientais.

**CONCLUSÕES:**

A associação de vários instrumentos requer tempo e dificulta o uso da classificação. A elaboração de instrumentos com associação direta às suas categorias se faz essencial para operacionalizá-la.

## INTRODUÇÃO

O uso de instrumentos de classificação na área da saúde ocupacional facilita a compreensão sobre os diferentes agravos e incapacidades advindos do trabalho ^1 , 2^ , já que suas consequências podem ser amplamente variadas. A padronização da linguagem sobre o estado de saúde dos trabalhadores otimiza as relações entre as diversas áreas que compõem a equipe de intervenção, possibilitando ações integradas e efetivas de retorno ao trabalho ^1^ .

A Organização Mundial de Saúde (OMS) propõe duas classificações para registrar informações sobre as condições e estados de saúde das populações. A primeira é a Classificação Estatística Internacional de Doenças e Problemas Relacionados com a Saúde (CID), que codifica as condições de saúde em termos de sinais e sintomas. A segunda é a Classificação Internacional de Funcionalidade, Incapacidade e Saúde (CIF), que classifica o impacto dessas condições em termos de funcionalidade ^4^ . Essas classificações devem ser utilizadas de forma conjunta, favorecendo a realização de comparações em pesquisas, registros de dados, alimentação de sistemas de informação, elaboração de relatórios e estatísticas em saúde pública ^5 , 6^ .

A reabilitação profissional (RP) é descrita como o principal processo para promover a saúde dos trabalhadores, objetivando manter o trabalhador ativo ou propiciando o seu retorno ao trabalho em casos de adoecimento ou incapacidade ^1 , 7 , 8^ . Deve ser realizada sob uma abordagem multiprofissional, com ações que vão desde a prevenção do adoecimento e assistência à saúde até a realização de alterações no ambiente de trabalho ^1 , 2 , 9^ .

No Brasil, a RP é historicamente atribuída ao Ministério da Previdência Social, tendo como critério central a identificação da incapacidade por meio da perícia com posterior concessão de benefício ao trabalhador afastado e encaminhamento a um programa de orientação profissional que objetive promover meios (treinamentos e cursos) para que o trabalhador seja reinserido no mercado de trabalho ^10^ . Não inclui, obrigatoriamente, ações integradas de reabilitação física e/ou psicológica, que são atribuições do Ministério da Saúde, ofertadas por centros de reabilitação, não necessariamente especializados nessa área de atuação ^10 , 11^ .

O *Manual Técnico de Procedimentos da Área da Reabilitação Profissional* , de 2016, aponta para um avanço nessa área, reconhecendo que a RP deve incluir ações combinadas de atenção, vigilância e assistência à saúde, incluindo a reinserção social e análise ambiental, com atuação de uma equipe multiprofissional especializada, sendo de responsabilidade pública e intersetorial e devendo ser pautada pela CIF em toda sua elaboração ^12^ . No entanto, a desarticulação entre os setores responsáveis e a falta de uma política pública consistente ainda contribuem para a não implantação de um programa de RP eficiente no Brasil, com muitos trabalhadores sem condições de retornar às suas atividades laborais ^10^ .

O *ICF Research Branch* é um importante centro colaborador da OMS para estudos com a CIF e é referência em publicações na área da RP. A adoção do modelo biopsicossocial da OMS, bem como o uso da CIF na RP, já são consolidados por permitir uma visão abrangente da incapacidade laboral e a padronização da linguagem, melhorando a comunicação entre profissionais de saúde, usuários, empregadores e gestores de políticas públicas, favorecendo os resultados esperados com a RP ^13^ .

Nesse sentido, um grupo de pesquisadores desse centro elaborou em 2012 o *core set* para RP, para servir de referência na descrição da funcionalidade de trabalhadores ^2^ . *Core set* é uma lista resumida de categorias da CIF, elaborada por consenso entre especialistas em determinada área, como uma estratégia proposta pela OMS para facilitar o uso da classificação entre os profissionais dos diversos setores ^14^ .

O *core set* para RP reúne 90 categorias da CIF e é aplicável a qualquer classe de trabalhadores, independentemente das condições de saúde. É dividido em 17 categorias do domínio de funções corporais, 40 categorias do domínio de atividades e participação e 33 categorias referentes aos fatores ambientais ( Tabela 1 ).

**Tabela 1:** Categorias do *core set* da Classificação Internacional de Funcionalidade, Incapacidade e Saúde para reabilitação profissional segundo Finger et al. 12

Funções corporais (b)		Atividades e participação (d)		Fatores ambientais (e)	
		
Categoria da CIF	Descrição da categoria	Categoria da CIF	Descrição da categoria	Categoria da CIF	Descrição da categoria
**b 117**	Funções intelectuais	d 155	Adquirir competências	e 1101	Medicamentos
**b 126**	Temperamento e personalidade	d 160	Concentrar atenção	e 115	Produtos para uso pessoal na vida diária
**b 130**	Energia e funções de impulso	d 163	Pensar	e 120	Produtos para mobilidade pessoal
**b 134**	Funções do sono	d 166	Ler	e 125	Produtos e tecnologias para comunicação
**b 140**	Funções da atenção	d 170	Escrever	e 130	Produtos e tecnologias para educação
**b 144**	Funções de memória	d 172	Calcular	e 135	Produtos e tecnologia para o emprego
**b 152**	Funções emocionais	d 175	Resolver problemas	e 150	Arquitetura, construção uso público
**b 160**	Funções do pensamento	d 177	Tomar decisões	e 155	Arquitetura, construção uso privado
**b 164**	Funções cognitivas de alto nível	d 210	Realizar uma única tarefa	e 225	Clima
**b 210**	Funções da visão	d 220	Realizar múltiplas tarefas	e 240	Luz
**b 230**	Funções da audição	d 230	Executar a rotina diária	e 250	Som
**b 235**	Funções vestibulares	d 240	Lidar com o stress e outras exigências psicológicas	e 260	Qualidade do ar
**b 280**	Sensação de dor	d 310	Comunicar e receber mensagens orais	e 310	Família imediata
**b 455**	Funções de tolerância ao exercício	d 315	Comunicar e receber mensagens não verbais	e 320	Amigos
**b 730**	Funções de força muscular	d 350	Conversar	e 325	Conhecidos, pares, colegas, vizinhos
**b 740**	Funções de *endurance* muscular	d 360	Utilizar dispositivos de comunicação	e 330	Pessoas em posição de autoridade
**b 810**	Funções de proteção da pele	d 410	Mudar a posição básica do corpo	e 340	Prestadores de cuidados pessoais
		d 415	Manter a posição do corpo	e 355	Profissionais de saúde
		d 430	Levantar e transportar objetos	e 360	Outros profissionais
		d 440	Utilizar movimentos finos da mão	e 430	Atitudes de pessoas em posição de autoridade
		d 445	Utilizar movimentos da mão e do braço	e 450	Atitudes individuais de profissionais de saúde
		d 450	Caminhar	e 460	Atitudes sociais
		d 455	Deslocar-se	e 465	Normas práticas e ideologias
		d 465	Deslocar-se usando algum equipamento	e 525	Serviços, sistemas e políticas de habitação
		d 470	Utilizar transporte	e 535	Serviços e políticas de comunicação
		d 475	Conduzir transporte	e 540	Serviços, e políticas de transportes
		d 530	Cuidados com o processo de excreção	e 550	Serviços, sistemas e políticas legais
		d 540	Vestir-se	e 555	Serviços e políticas de associações
		d 570	Cuidar da própria saúde	e 565	Serviços, sistemas e políticas econômicas
		d 710	Interações interpessoais básicas	e 570	Serviços, políticas de seguridade social
		d 720	Interações pessoais complexas	e 580	Serviços, sistemas e políticas de saúde
		d 740	Relacionamento formal	e 585	Serviços e políticas de educação e formação
		d 820	Educação escolar	e 590	Serviços e políticas de trabalho e emprego
		d 825	Formação profissional		
		d 830	Educação de nível superior		
		d 840	Estágio/preparação para o trabalho		
		d 845	Obter, manter e sair de um emprego		
		d 850	Trabalho remunerado		
		d 855	Trabalho não remunerado		
		d 870	Autossuficiência econômica		

No entanto, a CIF e seus *core sets* são instrumentos de classificação e, para que sejam acessados de forma fidedigna e seus achados passíveis de comparação entre profissionais e em pesquisas, são necessários métodos padronizados de medidas para avaliar a funcionalidade dos indivíduos e que sejam preferencialmente compatíveis com o uso da classificação ^14^ . Visando contribuir para a operacionalização da CIF, essencial para o avanço das políticas públicas de RP no Brasil, o presente estudo teve como proposta revisar os instrumentos de avaliação funcional e situação de saúde que são utilizados em pesquisas que avaliam funcionalidade e situação de saúde de trabalhadores brasileiros em geral e verificar a compatibilidade de seus itens com as categorias do *core set* para reabilitação profissional.

## MÉTODOS

Uma revisão da literatura foi realizada nas bases de dados PubMed, Scopus, Web of Science, Lilacs e SciELO *,* no período de janeiro a julho de 2018 *,* com objetivo de identificar instrumentos de avaliação da funcionalidade e situação de saúde aplicados em populações de trabalhadores brasileiros de qualquer categoria. A busca na literatura incluiu publicações no período de janeiro de 2007 a dezembro de 2017.

Os seguintes descritores foram considerados: *“capacity evaluation, work”, OR “disability evaluation, work”, AND “functional assessment”, OR “questionnaire”* . Os mesmos descritores foram utilizados para a busca em português.

Após a identificação dos artigos, foram selecionados os instrumentos utilizados nos estudos para a avaliação dos trabalhadores, e seus conteúdos foram pesquisados na íntegra. Em seguida, foram selecionados aqueles nos quais a maioria dos itens poderia responder às categorias do *core set* da CIF para RP, incluindo, preferencialmente, os com itens referentes à avaliação de alterações de funções corporais, limitação de atividades e questões ambientais, conforme propõe a classificação. A partir disso, foram feitas as análises dos itens de cada instrumento selecionado e verificada a possibilidade de acessar as categorias do *core set* da CIF para RP.

A compatibilidade de cada item dos instrumentos identificados na literatura, com o *core set,* foi verificada independentemente por dois avaliadores, que eram profissionais de saúde familiarizados com o uso da CIF, conforme recomendação da literatura ^15^ . Para essa análise, foi considerado o que o item contemplava e estabelecida a conexão com o *core set* por meio da descrição detalhada e das definições de cada categoria oferecidas pela CIF.

Para verificar a concordância entre os avaliadores na seleção das categorias acessadas pelos itens de cada instrumento, foram calculados os coeficientes kappa de Cohen, classificados pelos pontos de corte propostos por Landis e Koch ^16^: abaixo de 0 (pobre); 0 a 0,20 (fraca); 0,21 a 0,40 (razoável); 0,41 a 0,60 (moderada); 0,61 a 0,80 (substancial); e 0,81 a 1,00 (quase perfeita).

Após a análise da concordância, foi feito o estudo dos itens não concordantes entre os avaliadores em busca do consenso para retirada ou confirmação da inclusão do item como compatível com o *core set* .

## RESULTADOS

Foram encontrados 13 instrumentos de avaliação utilizados em pesquisas sobre funcionalidade ou estados de saúde de trabalhadores brasileiros no período estudado. Por meio da recuperação de seus conteúdos, constatou-se que todos foram validados especificamente para o Brasil para avaliação de aspectos relacionados à funcionalidade ou situação de saúde.

Dos treze instrumentos revisados, cinco foram criados especificamente para a avaliação de incapacidades e aspectos relacionados ao trabalho, sendo eles: o índice de capacidade para o trabalho (ICT), o *Cultural and Psychosocial Influences on Disability questionnaire* (CUPID), a *Work Disability Diagnosis Interview* (WoDDI), o *Obstacles to Return-to-Work Questionnaire* (ORTWQ) e o *Work Role Functioning Questionnaire* (WRFQ). Os demais foram instrumentos utilizados pelos pesquisadores, em grupos de trabalhadores, porém criados para avaliação específica de algumas regiões anatômicas ou para verificar a qualidade de vida em geral sem levar em conta a natureza e as condições de trabalho que podem estar associadas aos sintomas. Os instrumentos analisados e o número de categorias do *core set* para RP que puderam ser acessadas por cada um deles são apresentados na Tabela 2 .

**Tabela 2:** Instrumentos de avaliação funcional e situação de saúde selecionados para análise de compatibilidade com o *core set* da Classificação Internacional de Funcionalidade, Incapacidade e Saúde para reabilitação profissional e número de categorias do *core set* que puderam ser acessadas em cada domínio.

Instrumento	Autor, ano e local do estudo	Amostra de trabalhadores	Número de categorias acessadas		
			Funções corporais Total: 17	Atividades e participação Total: 40	Fatores ambientais Total: 33
ICT	Walsh et al., 2008. São Carlos, SP.	134 trabalhadoras de uma multinacional	3	9	0
CUPID	Carugno et al., 2012. São Paulo, SP.	751 enfermeiras de hospitais públicos	3	7	1
WoDDI	Mininel et al., 2012. São Paulo, SP.	30 trabalhadores do Hospital Universitário da USP	6	6	5
WRFQ	Galash e Costa, 2007. Campinas, SP.	105 trabalhadores (formais ou informais)	1	14	0
ORTWQ	Milani et al., 2016. Campinas, SP.	301 trabalhadores diversos	3	2	4
WHODAS	Valério et al., 2016. Uberaba, MG.	94 trabalhadores ativos (formais ou informais)	2	13	1
NHP	Bartilotti et al., 2009. Florianópolis, SC.	425 trabalhadores atendidos no CEREST de Santa Catarina	5	7	1
MIF	Bartilotti et al., 2009. Florianópolis, SC.	425 trabalhadores atendidos no CEREST de Santa Catarina	1	8	0
DASH	Camargo et al., 2007. São Carlos, SP.	27 trabalhadores industriais	4	5	0
ODI	Walsh et al., 2008. São Carlos, SP.	134 trabalhadoras de uma multinacional	2	6	0
RMQ	Sardá Jr et al., 2009. Florianópolis, SC.	234 trabalhadores de frigorífico	3	7	01
SF-36	Sena et al., 2013. Lagarto, SE.	351 trabalhadores rurais	3	10	0
WHOQOL-BREF	Ferreira et al., 2017. São Paulo, SP.	50 trabalhadores açougueiros	4	6	11

ICT: índice de capacidade para o trabalho; CUPID: *Cultural and Psychosocial Influences on Disability questionnaire* ; WoDDI: *Work Disability Diagnosis Interview* ; WRFQ: *Work Role Functioning Questionnaire* ; ORTWQ: *Obstacles to Return-to-Work Questionnaire* ; WHODAS: *World Health Organization Disability Assessment Schedule II* ; NHP: *Nottingham Health Profile* ; MIF: Medida de Independência Funcional; DASH: *Disabilities of the Arm, Shoulder and Hand questionnaire* ; ODI: *Owestry Low Back Pain Disability Questionnaire* ; RMQ: *Roland-Morris Questionnaire* ; SF-36: *Short Form (36) Health Survey* ; WHOQOL-BREF: versão reduzida do *World Health Organization Quality of Life questionnaire* ; USP: Universidade de São Paulo; CEREST: Centro de Referência em Saúde do Trabalhador

Entre esses instrumentos, quatro foram desenvolvidos com base nas categorias da CIF, o que permite uma maior conexão com ela: o *World Health Organization Disability Assessment Schedule II* (WHODAS), o *Nottingham Health Profile* (NHP), o *World Health Organization Quality of Life questionnaire* (WHOQOL) e o ORTWQ. O WHODAS apresentou concordância substancial entre os avaliadores no vínculo com o *core set* , e os demais concordância quase perfeita, conforme mostra a Tabela 3 . Os outros oito instrumentos revisados não foram criados com vistas à utilização da CIF, mas a concordância entre os itens escolhidos pelos avaliadores como compatíveis com as categorias do *core set* foi substancial ou quase perfeita para a maioria e moderada para o CUPID ( Tabela 3 ).

**Tabela 3:** Instrumentos analisados e valores de kappa encontrados na análise da concordância entre os avaliadores.

Instrumento	Valor do kappa
WRFQ	0,91
NHP	0,87
ODI	0,86
ORTWQ	0,83
DASH	0,82
WHOQOL-BREF	0,81
SF-36	0,78
WHODAS	0,74
RMQ	0,73
MIF	0,72
ICT	0,67
WoDDI	0,61
CUPID	0,49

WRFQ: *Work Role Functioning Questionnaire* ; NHP: *Nottingham Health Profile* ; ODI: *Owestry Low Back Pain Disability Questionnaire* ; ORTWQ: *Obstacles to Return-to-Work Questionnaire* ; DASH: *Disabilities of the Arm, Shoulder and Hand questionnaire* ; WHOQOL-BREF: versão reduzida do *World Health Organization Quality of Life questionnaire* ; SF-36: *Short Form (36) Health Survey* ; WHODAS: *World Health Organization Disability Assessment Schedule II* ; RMQ: *Roland-Morris Questionnaire* ; MIF: Medida de Independência Funcional; ICT: índice de capacidade para o trabalho; WoDDI: *Work Disability Diagnosis Interview* ; CUPID: *Cultural and Psychosocial Influences on Disability questionnaire*

Nota: Classificação segundo Landis e Koch ^16^: pobre (<0), fraca (0 a 0,20), razoável (0,21 a 0,40), moderada (0,41 a 0,60), substancial (0,61 a 0,80), quase perfeita (0,81 a 1).

A análise da compatibilidade dos itens dos instrumentos selecionados com o *core set* para RP evidenciou a variabilidade no enfoque de cada instrumento ao abordar as questões funcionais do indivíduo quando o objetivo é capacidade ocupacional. A proporção de categorias do *core set* acessadas pelos itens de cada questionário de acordo com os componentes da CIF é apresentada no Gráfico 1 e nota-se a ênfase dada à avaliação de funções corporais e de limitação de atividades e participação. Os instrumentos específicos para avaliação de aspectos relacionados ao trabalho (ICT, CUPID, WoDDI, WRFQ e ORTWQ), incluindo limitações funcionais, puderam acessar, quando analisados em conjunto, 36 categorias do *core set* para reabilitação profissional (11 categorias b, 15 categorias d e 10 categorias e).

**Gráfico 1:** Proporção de categorias preenchidas pelos instrumentos analisados em cada domínio do *core set* para reabilitação profissional. ICT: índice de capacidade para o trabalho; CUPID: *Cultural and Psychosocial Influences on Disability questionnaire* ; WoDDI: *Work Disability Diagnosis Interview* ; WRFQ: *Work Role Functioning Questionnaire* ; ORTWQ: *Obstacles to Return-to-Work Questionnaire* ; DASH: *Disabilities of the Arm, Shoulder and Hand questionnaire* ; ODI: *Owestry Low Back Pain Disability Questionnaire* ; MIF: Medida de Independência Funcional; NHP: *Nottingham Health Profile* ; RMQ: *Roland-Morris Questionnaire* ; SF-36: *Short Form (36) Health Survey* ; WHODAS: *World Health Organization Disability Assessment Schedule II* ; WHOQOL-BREF: versão reduzida do *World Health Organization Quality of Life questionnaire*

O ICT tem como objetivo evidenciar quão bem está ou estará um trabalhador num futuro próximo e quão bem ele é capaz de executar seu trabalho em função das exigências, de seu estado de saúde e de suas capacidades físicas e mentais. Inclui domínios sobre a capacidade atual para o trabalho, capacidade em relação às exigências do trabalho, número de doenças diagnosticadas, perda estimada da capacidade para o trabalho e falta por causa das doenças, prognóstico da capacidade para o trabalho daqui a dois anos e recursos mentais. Pelos seus itens foi possível estabelecer conexão com 12 categorias do *core set* em questão.

O CUPID objetiva associar os sintomas musculoesqueléticos dos trabalhadores às suas atividades, aspectos psicossociais e outras incapacidades. Validado para o Brasil como Pesquisa Internacional sobre as Influências Físicas, Culturais e Psicossociais nos Sintomas Musculoesqueléticos e Incapacidades Associadas, verifica as atividades físicas no trabalho, aspectos psicossociais, sintomas musculoesqueléticos em vários sítios anatômicos associados à incapacidade para tarefas diárias comuns, saúde mental e tendência para se preocupar com os sintomas, bem como crenças sobre a natureza e a gravidade das doenças relacionadas ao trabalho. Esse instrumento possibilitou conexão com 11 categorias.

O WoDDI busca detectar os fatores preditivos de maior importância para incapacidades relacionadas ao trabalho e identificar uma ou mais causas de absenteísmo prolongado. Validado para o Brasil como Instrumento de Identificação da Situação de Incapacidade para o Trabalho, analisa a história da moléstia atual, dor, condição de saúde anterior e atual, exame físico, hábitos de vida, história sociofamiliar, situação financeira, ambiente de trabalho, percepções do trabalhador e análise de resultados e recomendações. Seus itens foram compatíveis com 17 categorias do *core set* .

O WRFQ é validado para o português como Questionário de Avaliação do Desempenho no Trabalho e avalia se a capacidade funcional do trabalhador está alterada devido a problemas de saúde. Analisa a demanda de trabalho, demanda física, demanda mental, demanda social e demanda de produção. Teve conexão com 15 categorias.

O último instrumento voltado à avaliação de trabalhadores, o ORTWQ, foi criado sob influência da CIF e avalia barreiras relacionadas ao retorno ao trabalho. Validado para o Brasil como Obstáculos para Retorno ao Trabalho, contém 55 itens divididos em nove domínios: dificuldade de retorno, carga física e autopercepção da nocividade do trabalho, suporte social, preocupação devido ao afastamento, satisfação, suporte/situação familiar e prognóstico autopercebido de retorno ao trabalho. Apesar de extenso, a análise de seus itens permitiu verificar uma conexão com apenas nove categorias do *core set* .

Os demais instrumentos revisados foram utilizados nas pesquisas de forma adaptada para estabelecimento de problemas em trabalhadores e, quando vinculados ao *core set* para reabilitação profissional, também apresentaram variabilidade nas categorias respondidas, com ênfase em funções corporais e limitação de atividades. Utilizados em conjunto, esses instrumentos puderam acessar 45 categorias, sendo sete relacionadas a funções corporais, 24 a atividades e participação e 13 a fatores ambientais.

O WHODAS foi criado pela OMS para oferecer correlação direta com a CIF e servir como um método padronizado de mensuração da saúde e das incapacidades de forma transcultural. Contém 36 itens e fornece o nível de funcionalidade em seis domínios: cognição, mobilidade, autocuidado, relações interpessoais, atividades de vida e participação. Analisado para acessar o *core set* , foi possível estabelecer conexão com 16 categorias.

O NHP é um instrumento genérico com 38 itens que se referem aos seguintes domínios: nível de energia, dor, reações emocionais, sono, interação social e habilidades físicas. Objetiva avaliar a qualidade de vida em pacientes portadores de doenças crônicas. A análise dos seus itens mostrou compatibilidade com 13 categorias.

A *Functional Independence Measure* (FIM), validada para o português como escala de Medida de Independência Funcional (MIF), foi elaborada com base nas categorias da versão-teste da CIF chamada de *International Classification of Impairments, Disabilities, and Handicaps* (ICIDH). Validada para uma ampla categoria de pessoas no Brasil, mensura o nível de incapacidade do indivíduo e o quanto de assistência é requerida para realizar suas atividades. Conta com 18 itens distribuídos nos seguintes domínios: autocuidado, controle de esfíncteres, mobilidade, locomoção, comunicação e interação social. Foi compatível com 18 categorias do *core set.*

Outro instrumento utilizado na avaliação funcionalidade em trabalhadores é o *Disabilities of the Arm, Shoulder and Hand questionnaire* (DASH), criado para avaliação de disfunção e sintomas físicos em membros superiores em qualquer grupo de pessoas. Inclui 30 itens relacionados a dor, fraqueza, rigidez, formigamento, atividades diárias, tarefas domésticas, compras, atividades de recreação, autocuidado, vestir, alimentação, atividades sexuais, sono, cuidados com a família, trabalho, socialização e autoimagem. Em relação ao *core set* , o DASH permitiu acessar nove categorias.

O *Owestry Low Back Pain Disability Questionnaire* (ODI) e o *Roland-Morris Questionnaire* (RMQ) também são utilizados para avaliação de capacidade funcional. Ambos validados para o Brasil em diversos tipos populacionais, avaliam o efeito da lombalgia na funcionalidade. Tiveram conexão com 8 e 11 categorias do *core set* , respectivamente.

Os dois últimos instrumentos revisados avaliam a qualidade de vida dos indivíduos, considerando aspectos funcionais. O *Short Form (36) Health Survey* (SF-36) avalia a qualidade de vida dos indivíduos e inclui questões referentes à funcionalidade. É genérico e aborda os seguintes aspectos: capacidade funcional, aspectos físicos, dor, estado geral de saúde, vitalidade, aspectos sociais, aspectos emocionais, saúde mental e avaliação comparativa de saúde. Possibilitou estabelecer conexão com 13 categorias.

O WHOQOL-BREF também avalia qualidade de vida. É uma versão reduzida do WHOQOL, proposto pela OMS para ser utilizado de forma transcultural, composto por 26 itens distribuídos em quatro domínios: físico, psicológico, relações sociais e meio ambiente. Possibilitou o vínculo a 21 categorias.

Conforme este estudo, para avaliarmos todos os aspectos considerados relevantes apontados nas categorias do *core set* para reabilitação profissional, seria necessária a utilização de uma combinação de instrumentos. O preenchimento do máximo de categorias do *core set* utilizando o mínimo de instrumentos possíveis demandaria a utilização de 10 dos 13 revisados neste trabalho e seria possível acessar 58 das 90 categorias propostas ( Tabela 4 ). Entre elas, 13 (76%) seriam referentes ao componente de funções corporais, 28 (72,5%) ao de atividades e participação e 16 (49%) ao de fatores ambientais.

**Tabela 4:** Relação das categorias do *core set* para reabilitação profissional acessadas pelos dez instrumentos de avaliação selecionados como mínimos para responder ao *core set.*

Categorias*	Instrumentos
b 126: funções de temperamento	RMQ, DASH, WRFQ
b 130: energia e funções de impulso	ICT, ORTWQ, SF-36, WHOQOL-BREF
b 134: funções do sono	ICT, WoDDI, RMQ, DASH, WHOQOL-BREF
b 140: funções da atenção	ICT
b 144: funções de memoria	WHODAS
b 152: funções emocionais	CUPID, ICT, ORTWQ, WoDDI, SF-36, WHODAS, WHOQOL-BREF
b 210: funções da visão	ICT
b 230: funções da audição	ICT
b 235: funções vestibulares	CUPID
b 280: sensação de dor	CUPID, ICT, ORTWQ, WoDDI, RMQ, DASH, SF-36, WHOQOL-BREF
b 455: funções de tolerância ao exercício	ICT, WoDDI
b 730: funções de força muscular	WoDDI, DASH
b 810: funções de proteção da pele	ICT, WoDDI
d 160: concentrar atenção	WHODAS, WHOQOL-BREF, WRFQ
d 163: pensar	WRFQ
d 166: ler	WRFQ
d 170: escrever	CUPID, DASH
d 175: resolver problemas	ICT, WHODAS
d 177: tomar decisões	ICT
d 210: realizar uma única tarefa	ORTWQ, WHODAS, WRFQ
d 220: realizar múltiplas tarefas	RMQ
d 230: executar a rotina diária	CUPID, RMQ, SF-36, WHODAS, WHOQOL-BREF
d 310: comunicar (mensagens orais)	WHODAS, WRFQ
d 350: conversação	WHODAS
d 360: utilização de dispositivos de comunicação	WRFQ
d 410: mudar a posição básica do corpo	CUPID, RMQ, SF-36, WHODAS, WRFQ
d 415: manter a posição do corpo	RMQ, WHODAS, WRFQ
d 430: levantar e transportar objetos	WoDDI, DASH, SF-36, WRFQ
d 440: utilizar movimentos finos da mão	DASH, CUPID, WRFQ
d 445: utilização da mão e do braço	DASH, CUPID, ORTWQ, SF-36, WRFQ
d 450: caminhar	CUPID, WoDDI, RMQ, SF-36, WHODAS, WHOQOL-BREF, WRFQ
d 455: deslocar-se	SF-36
d 465: deslocar-se com equipamento	WoDDI, RMQ
d 470: utilização de transporte	DASH
d 530: cuidados com processo de excreção	MIF
d 540: vestir-se	CUPID, RMQ, DASH, SF-36, WHODAS
d 570: cuidar da própria saúde	WHODAS
d 710: interações interpessoais básicas	SF-36, WHODAS, WHOQOL-BREF
d 740: relacionamento formal	WoDDI, WRFQ
d 845: obter, manter e sair de um emprego	SF-36
d 850: trabalho remunerado	ICT, WoDDI, SF-36, WHODAS, WHOQOL-BREF, WRFQ
d 870: autossuficiência econômica	WoDDI, WHOQOL-BREF
e 1101: medicamentos	WoDDI
e 120: produtos de mobilidade	RMQ
e 150: arquitetura (prédios de uso público)	WHODAS
e 225: clima	WoDDI, WHOQOL-BREF
e 240: luz	WHOQOL-BREF
e 250: som	WoDDI, WHOQOL-BREF
e 260: qualidade do ar	WoDDI, WHOQOL-BREF
e 310: família imediata	ORTWQ, WHOQOL-BREF
e 320: amigos	ORTWQ, WHOQOL-BREF
e 325: conhecidos	ORTWQ, WoDDI, WHOQOL-BREF
e 330: pessoas em posição de autoridade	CUPID
e 355: profissionais de saúde	WHOQOL-BREF
e 430: atitudes de pessoas em posição de autoridade	ORTWQ
e 525: serviços relacionados à habitação	WHOQOL-BREF
e 540: serviços relacionados à transportes	WHOQOL-BREF
e 580: serviços de saúde	WoDDI, WHOQOL-BREF

WRFQ: *Work Role Functioning Questionnaire* ; NHP: *Nottingham Health Profile* ; ODI: *Owestry Low Back Pain Disability Questionnaire* ; ORTWQ: *Obstacles to Return-to-Work Questionnaire* ; DASH: *Disabilities of the Arm, Shoulder and Hand questionnaire* ; WHOQOL-BREF: versão reduzida do *World Health Organization Quality of Life questionnaire* ; SF-36: *Short Form (36) Health Survey* ; WHODAS: *World Health Organization Disability Assessment Schedule II* ; RMQ: *Roland-Morris Questionnaire* ; MIF: Medida de Independência Funcional; ICT: índice de capacidade para o trabalho; WoDDI: *Work Disability Diagnosis Interview* ; CUPID: *Cultural and Psychosocial Influences on Disability questionnaire*

Nota: 32 categorias do *core set* , descritas na Tabela 1, não foram identificadas em nenhum instrumento, sendo quatro de funções corporais (b117, b160, b164, b740), 11 de atividades e participação (d155, d172, d240, d315, d475, d720, d820, d825, d830, d840, d855) e 17 de fatores ambientais (e115, e125, e130, e135, e155, e340, e360, e450, e460, e465, e535, e550, e565, e555, e570, e585, e590).

Analisados para responder ao *core set* para RP, a maioria dos instrumentos prioriza a avaliação de alterações em funções corporais e a identificação de limitação de atividades que possam interferir no trabalho. Os fatores ambientais são considerados por seis instrumentos, ou seja, a interferência do meio ambiente como facilitador ou limitador da funcionalidade é pouco abordada pela maioria dos instrumentos levantados.

## DISCUSSÃO

O presente estudo buscou analisar instrumentos de avaliação funcional como forma de acessar o *core set* da CIF para RP, visando melhorar a compreensão sobre as medidas de funcionalidade nessa área e elucidar possíveis formas de utilização da classificação. Esse *core set* foi escolhido por ser o único, na literatura, criado para nortear a classificação da funcionalidade de trabalhadores em reabilitação, por meio da CIF. Para utilizá-lo é importante a aplicação de instrumentos validados que mensurem a funcionalidade de forma fidedigna, gerando resultados passíveis de comparações em pesquisas ^2^ .

A revisão realizada durante a pesquisa evidenciou a multiplicidade de instrumentos utilizados na abordagem de questões funcionais relacionadas ao trabalho. Foram elegíveis aqueles que pudessem alcançar os domínios da CIF, respondendo ao máximo de categorias do *core set* estudado. Dessa forma foram selecionados cinco instrumentos específicos e oito instrumentos genéricos que avaliaram a funcionalidade de trabalhadores publicados no período proposto.

A grande variedade de instrumentos disponíveis, com diferentes bases teóricas, leva à necessidade de escolha por parte dos pesquisadores e dificulta a comparação e padronização dos resultados ^17^ . Uma forma de compensar essa lacuna é vincular diferentes instrumentos de avaliação a um modelo conceitual único que favoreça um quadro comum para comparar medidas ^18^ .

A CIF é uma base conceitual teórica, endossada pela OMS, para classificar a funcionalidade e incapacidade dos indivíduos e promover uma linguagem única e abrangente sobre o estado de saúde das populações ^21 , 22^ . Vinculada a instrumentos de avaliação, a classificação permite a tradução das medidas para um mesmo idioma (códigos da CIF), facilitando a análise de conteúdo de seus itens e a compreensão e comparação dos resultados ^23^ .

Analisados tendo como referência o *core set* para RP, foi possível perceber que os instrumentos reúnem itens em comum, mas diferem na abordagem dos domínios considerados pela CIF. Cerca de 38% das categorias do *core set* puderam ser acessadas por mais de um dos 13 instrumentos. Em relação aos domínios, os componentes relacionados a problemas em funções corporais e limitação de atividades e participação foram os mais representados, evidenciando a preocupação em verificar o adoecimento físico e a experiência dos trabalhadores nas diversas atividades envolvidas em um modo de trabalho.

Trinta e duas das 90 categorias do *core set* não puderam ser acessadas por meio dos itens dos instrumentos encontrados. O componente de funções corporais teve quatro categorias (23%) não acessadas, o de atividades e participação 11 categorias (27,5%) e o componente menos representado foi de fatores ambientais, com 17 categorias (51,5%) sem respostas.

Outros autores também verificaram essa menor representatividade de itens relacionados às categorias de fatores ambientais da CIF em correlações com outros instrumentos ^17 , 19 , 24^ . Esse fato desfavorece a abordagem dada à funcionalidade, já que ignora um importante componente influenciador na função dos indivíduos, altamente considerado pelo modelo biopsicossocial que embasou a elaboração da CIF ^23 , 27^ .

Na RP os fatores ambientais (físicos, sociais e atitudinais) devem ser enfatizados, já que são importantes influenciadores da participação no trabalho, interagindo com as condições do corpo (funções e estruturas corporais) e determinando o nível e a extensão do seu funcionamento ^1 , 28^ . O objetivo principal desse processo é a recuperação da capacidade laboral de forma eficaz e duradoura, o que está intimamente associado às condições do local de trabalho ^3^ .

Para se manter ativo e produtivo, o trabalhador precisa estar em um ambiente propício, que deve incluir facilitadores da funcionalidade, desde condições ergonômicas adequadas até mudanças organizacionais geradoras de bem-estar, aumento da autoestima, autonomia no trabalho e relacionamentos saudáveis ^1 , 29^ . Mesmo indivíduos altamente incapacitados podem ter suas possibilidades de participação recuperadas se a modificação do ambiente estiver entre as prioridades da RP ^1 , 3^ . Esse processo se dá com a análise dos fatores ambientais que devem ser cuidadosamente inseridos na avaliação e no acompanhamento de cada trabalhador ^3^ .

O coeficiente kappa, utilizado para verificar a concordância entre os avaliadores que analisaram a correspondência dos itens dos instrumentos com as categorias do *core set* , variou de concordância moderada (k = 0,49) para um instrumento até quase perfeita (k > 0,80) para sete instrumentos ( Tabela 3 ). Esse resultado indica que, apesar de os instrumentos não oferecerem ligação direta com a CIF, os avaliadores tiveram entendimento comum na escolha da maioria das categorias acessadas por cada item.

Resultados semelhantes foram encontrados em outros estudos na ligação da CIF com diferentes ferramentas, e os autores ressaltam que a discordância entre os avaliadores pode se dever à CIF apresentar categorias mais específicas em algumas áreas do que em outras ^19 , 22 , 27^ . Além disso, a interpretação de determinado conceito presente em um item de um instrumento pode diferir entre os avaliadores, de forma que categorias distintas do *core set* sejam selecionadas, fato que justifica a necessidade do consenso na escolha final das categorias acessadas pelos questionários ^30^ . O vínculo de instrumentos de avaliação com a CIF permite uma análise padronizada a respeito de seus conteúdos, favorecendo a escolha do instrumento mais adequado para uso na prática clínica ^25 , 30^ .

Conforme este estudo, a avaliação de todos os aspectos considerados relevantes na funcionalidade do trabalhador, sugeridas no *core set* para reabilitação profissional, demandaria a combinação de diversos instrumentos e a busca por mais formas de avaliação para incluir os itens faltantes, já que não foram contempladas todas as categorias. Esse resultado é aceitável, já que os *core sets* são instrumentos criados por uma metodologia de consenso entre especialistas que buscam reunir categorias da CIF voltadas a grupos específicos da população, baseados no conhecimento e experiência clínica na área, sem levar em conta os instrumentos de avaliação disponíveis para acessá-los ^31^ .

Para que a CIF e seus *core sets* sejam utilizados com mais praticidade e de maneira uniforme, recomenda-se a criação de instrumentos de medidas sintonizados com a classificação ^34^ . Para isso, os autores deste *core set* criaram o *Work Rehabilitation Questionnaire* (WORQ) ^34^ , adaptado para o Brasil como Questionário de Reabilitação para o Trabalho, que pode ser empregado para avaliar parte das categorias, proporcionando vínculo direto com a CIF.

Este estudo é inovador ao analisar um número considerável de instrumentos que têm sido aplicados na avaliação da funcionalidade de trabalhadores e verificar suas abordagens tendo como referência um *core set* da CIF específico para essa área de atuação. O fato de não ter sido analisada a totalidade de instrumentos disponíveis para avaliação funcional incluindo aspectos físicos ou psíquicos e que podem ser utilizados com trabalhadores não compromete o resultado do estudo, já que seu objetivo foi ilustrar a operacionalização da CIF por meio deste *core set* específico, priorizando instrumentos que pudessem responder ao maior número de categorias possível.

Nesse sentido observou-se que não foi possível estabelecer vinculação de itens com a totalidade das categorias do *core set* quando são utilizados instrumentos criados sem o propósito de utilização da classificação. Outros estudos são necessários para verificar o grau de compatibilidade entre o modo como cada ferramenta quantifica a magnitude do comprometimento avaliado e o modo adotado pela CIF por meio de seus qualificadores, fato que pode se constituir em mais uma barreira para a classificação da funcionalidade avaliada a partir destes instrumentos.

Além disso, o vínculo a uma mesma base conceitual possibilitou a visualização dos aspectos comuns existentes entre os instrumentos estudados, bem como das diferenças na forma de abordar a funcionalidade ou o estado de saúde dos indivíduos.

Ficou claro que os fatores ambientais ainda são altamente desconsiderados pelos modelos de avaliação da funcionalidade utilizados nas pesquisas com trabalhadores. Isso é negativo quando se objetiva restaurar a capacidade laboral e reinserir o indivíduo no seu local de trabalho, pois os fatores ambientais são determinantes para a efetividade das ações e manutenção da funcionalidade. É necessário incluir a avaliação do ambiente no processo da RP, e isso pode ser feito com a utilização da CIF, tendo como referência as categorias ambientais sugeridas pelo *core set* estudado.

## CONCLUSÃO

A revisão de instrumentos de avaliação de funcionalidade e situação de saúde de trabalhadores e o posterior vínculo com o *core set* para reabilitação profissional realizados por este estudo concluiu que pelo menos dez instrumentos seriam necessários para avaliar 65% dos aspectos considerados relevantes nas categorias do *core set* para reabilitação profissional. O componente de fatores ambientais do *core set* foi o que apresentou menor possibilidade de respostas por meio dos itens dos questionários estudados, indicando a baixa ênfase dada à influência destes fatores na geração de incapacidades. A associação de vários instrumentos para responder a um *core set* específico requer tempo e dificulta a utilização da classificação. Instrumentos de avaliação elaborados para permitir associação direta às categorias e aos qualificadores da CIF se fazem essenciais para operacionalizá-la.
